# Epitope profiling of monoclonal antibodies to the immunodominant antigen BmGPI12 of the human pathogen *Babesia microti*

**DOI:** 10.3389/fcimb.2022.1039197

**Published:** 2022-11-25

**Authors:** Meenal Chand, Jae-Yeon Choi, Anasuya C. Pal, Pallavi Singh, Vandana Kumari, Jose Thekkiniath, Jacqueline Gagnon, Sushma Timalsina, Gauri Gaur, Scott Williams, Michel Ledizet, Choukri Ben Mamoun

**Affiliations:** ^1^ Department of Internal Medicine, Section of Infectious Diseases, Yale School of Medicine, New Haven, CT, United States; ^2^ L2 Diagnostics, LLC, New Haven, CT, United States; ^3^ Department of Forestry and Horticulture, Connecticut Agricultural Experiment Station, New Haven, CT, United States

**Keywords:** human babesiosis, apicomplexan parasite, *Babesia microti*, antigen capture, ELISA, BmGPI12, antibodies, epitope mapping

## Abstract

The significant rise in the number of tick-borne diseases represents a major threat to public health worldwide. One such emerging disease is human babesiosis, which is caused by several protozoan parasites of the *Babesia* genus of which *B. microti* is responsible for most clinical cases reported to date. Recent studies have shown that during its intraerythrocytic life cycle, *B. microti* exports several antigens into the mammalian host using a novel vesicular-mediated secretion mechanism. One of these secreted proteins is the immunodominant antigen BmGPI12, which has been demonstrated to be a reliable biomarker of active *B. microti* infection. The major immunogenic determinants of this antigen remain unknown. Here we provide a comprehensive molecular and serological characterization of a set of eighteen monoclonal antibodies developed against BmGPI12 and a detailed profile of their binding specificity and suitability in the detection of active *B. microti* infection. Serological profiling and competition assays using synthetic peptides identified five unique epitopes on the surface of BmGPI12 which are recognized by a set of eight monoclonal antibodies. ELISA-based antigen detection assays identified five antibody combinations that specifically detect the secreted form of BmGPI12 in plasma samples from *B. microti*-infected mice and humans but not from other *Babesia* species or *P. falciparum*.

## Introduction

Recent epidemiological reports have raised concerns about the rapid increase in tick-borne diseases in the United States and worldwide and the threat they pose to public health (Rosenberg et al., 2018; Kumar et al., 2021; Renard and Ben Mamoun, 2021). Major factors influencing this rise include anthropogenic alterations of the ecosystem, socio-economic changes, and climate change, as well as improved diagnostic methods and better notification (Gilbert, 2021). More than 75% of all vector-borne related illnesses reported in the United States between 2004 and 2016 were caused by tick-borne pathogens (Rosenberg et al., 2018; Renard and Ben Mamoun, 2021).

One such emerging tick-borne disease is human babesiosis, which has a global distribution and remains an endemic disease in the United States (Renard and Ben Mamoun, 2021). The disease is caused by several protozoan parasites of the *Babesia* genus (Renard and Ben Mamoun, 2021; Pal et al., 2022). Depending on the pathogen and the host immune status, the infection can lead to moderate or severe life-threatening illness, especially among immunocompromised hosts such as asplenic, cancer, and HIV patients, individuals on immunosuppressive drugs such as Rituximab, neonates as well as individuals over the age of 50 (Kumar et al., 2021).

Of the 8 species of *Babesia* that are associated with documented clinical reports of human babesiosis worldwide, *Babesia microti* accounts for the vast majority of mild, complicated or fatal clinical cases (Renard and Ben Mamoun, 2021). This eukaryotic pathogen is a member of the *Apicomplexa* phylum which also includes *Plasmodium* parasites, the causative agents of human malaria (Renard and Ben Mamoun, 2021). *Ixodes scapularis* ticks, which also transmit Lyme disease, anaplasmosis and Powassan encephalitis, are the main vectors of *B. microti*. However other modes of transmission have also been reported and include blood transfusion, organ transplantation, and perinatal transmission (Bloch et al., 2021; Krause et al., 2021; Meredith et al., 2021; Renard and Ben Mamoun, 2021). Approved treatment for human babesiosis include the use of drug combinations consisting of atovaquone and azithromycin for mild disease, and quinine and clindamycin for severe disease (Krause et al., 2021; Renard and Ben Mamoun, 2021). The emergence of drug-resistant parasites following treatment, and the commonly reported side effects associated with some of these drugs have led to the use of other antimalarial drugs such as proguanil and tafenoquine in the treatment of human babesiosis (Vyas et al., 2007; Carvalho et al., 2020). More specific drugs tailored to *Babesia* parasites, which have been evaluated in animal models of human babesiosis, have also been developed and include endochin-like quinolones ELQ-502, ELQ-331 and ELQ468, which alone or in combination with atovaquone have shown strong efficacy (Chiu et al., 2021; Chiu et al., 2022; Pal et al., 2022).

Following tick injection of *B. microti* sporozoites, the parasites invade human red blood cells, develop into mature ring and filamentous forms, and then divide to produce 4 daughter parasites per infected erythrocyte (Thekkiniath et al., 2018). Unlike *P. falciparum*, *B. microti* does not degrade hemoglobin and relies heavily on host plasma as a nutrient source for survival (Cornillot et al., 2012; Silva et al., 2016). Recent studies have shown that *B. microti* uses a unique mechanism of protein secretion to deliver its proteins into the host (Thekkiniath et al., 2019). Electron microscopy analysis showed that during its intraerythrocytic cycle, the parasite produces a string of attached vesicles that emerge out of the parasite plasma membrane and contain most of the parasite cytoplasm including proteins and ribosomes (Thekkiniath et al., 2019). Immunoproteomic analyses, using sera from *B. microti*-infected mice and humans, identified several antigens whose IgM and IgG profiles correlate with the early and late stages of infection, respectively (Cornillot et al., 2016; Silva et al., 2016). Interestingly, several of these immune-reactive antigens have been found to be associated with these vesicles and include BmGPI12, a member of the BMN1 multigene family of antigens, and BmIPA48, a constituent of the rhoptry organelle (Thekkiniath et al., 2019; Bastos et al., 2021). Transcription analyses showed that *BmGPI12* is one of the most highly expressed genes of *B. microti* (Cornillot et al., 2012; Carpi et al., 2016; Silva et al., 2016). Fractionation and immunoelectron microscopy studies have shown that BmGPI12 protein can be found in three major fractions: the parasite fraction where the protein was found to be associated with the parasite plasma membrane; the red blood cytoplasm fraction; and the extracellular fraction where the protein is readily detected by immunoblot and ELISA assays (Thekkiniath et al., 2018). The unique profile of this protein has made it an excellent choice for detection of active *B. microti* infection (Thekkiniath et al., 2018).

In this study, we aimed to characterize the epitope map of eighteen monoclonal antibodies developed against BmGPI12 (Gagnon et al., 2022) with the goal to develop specific antigen capture ELISA-based assays for detection of active *B. microti* infection. Our study identified five antibody combinations that specifically detect secreted BmGPI12 in *B. microti*-infected plasma but not in plasma from *B. duncani*-infected mice or supernatants of *B. duncani*, *B. divergens* and *P. falciparum in vitro* cultures in human red blood cells.

## Materials and methods

### Ethics statement

All animal experiments were approved by the Institutional Animal Care and Use Committees (IACUC) at Yale University (Protocol #2020-07689). Animals were acclimatized for one week after arrival before the start of an experiment. Animals that showed signs of distress or appeared moribund were humanly euthanized using approved protocols.

### Parasite strains and mouse infections

5-6 weeks old female C.B-17.SCID (Severe Combined immunodeficient) (C.B-17/IcrHsd-Prkdc^scid^) mice obtained from Envigo, NJ, and 5-6 weeks old female Balb/cJ mice purchased from Jackson Laboratories were used in this study. Mouse infections were established by intravenous injection of previously cryo-preserved *B. microti* (LabS1 strain)-infected blood. For studies involving *B. duncani*-infected mice, C3H/HeJ mice from Jackson Laboratories were infected with 10^6^-*B. duncani* (WA-1) red blood cells as previously described (Pal et al., 2022).

### BmGPI12 monoclonal antibodies

The anti-BmGPI12 monoclonal antibodies (MAbs) described in this study have been generated and purified as described by Gagnon and colleagues (Gagnon et al., 2022). These antibodies and the concentration used in this study are listed in **Table 1**.

**Table 1:** List of BmGPI12 mouse monoclonal antibodies and their concentrations.

BmGPI12 MAb	Concentration (μg/mL)
1A5	1030
1B10	3160
1E11	1070
1G11	2310
2A7	1789
2H6	2135
3A12	2400
3B1	1850
3B6	1020
3D4	2575
4B12	995
4C8	1090
4C12	350
4E1	1517
4F8	1648
4H5	1430
5C11	840
5D11	2545

### Production of recombinant sub fragments of BmGPI12

Codon-optimized DNA fragments encoding the full length BmGPI12 and sub-fragments F1 (amino acids 1-100), F2 (amino acids 50-150), F3 (amino acids 100 to 200), F4 (amino acids 150 to 250) and F5 (amino acids 200 to 328) were synthesized (Genscript Inc.), cloned into the *BamHI* and *XhoI* sites of the pGEX-6p expression plasmid (GE Healthcare) and the resulting plasmids were introduced into *E. coli* BL21 strain. Expression and purification of the recombinant proteins was conducted as described previously (Thekkiniath et al., 2018) with some modifications. Briefly, the *E. coli* strains harboring the plasmids were pre-grown overnight in Luria-Bertani (LB) Broth containing ampicillin (100 µg/ml). The cells were diluted by 100-fold in 200 ml of fresh medium and grown to OD_600_ of 0.5. Expression of the recombinant proteins was induced by adding 0.1 mM isopropyl-β-d-thiogalactopyranoside (IPTG) (I56000-25.0, RPI) to the cell culture and continued by incubation for 5h at 30°C while shaking at 230 rpm. Cells were spun down by centrifugation at 2,900 x g for 20 min at 4°C and the pellets were re-suspended in 5 ml of phosphate buffered saline (PBS, pH 7.3) buffer supplemented with protease inhibitor cocktail (Mini-cOmplete™ EDTA-free (11836170001, Roche). The cell free extracts were obtained by sonication using an Omni Sonic Ruptor 400 Ultrasonic Homogenizer (15 sec burst at 30% amplitude, 10 times, with 15 sec cooling on ice after each burst), followed by centrifugation at 16,000 g for 20 min. GST tagged recombinant proteins were purified by affinity chromatography on a 1 ml of glutathione sepharose high performance column (17-5279-01, Cytiva) by following the manufacturer’s instructions (Cytiva, 71502754 AC). Proteins bound to the column were eluted by applying 1 ml of elution buffer (50 mM Tris-HCl, 100 mM NaCl, 5 mM DTT, and 10 mM reduced glutathione, pH 8.0) to the column 3 times and glutathione was removed from the purified protein solutions using PD-10 desalting columns containing Sephadex G-25 resin (17085101, Cytiva). The purified proteins were used in the western blot, dot blot and ELISA assays described in this study.

### BmGPI12 peptide library

A library of 19 overlapping peptides covering a portion of the BmGPI12 protein between amino acids 50 and 200 were synthesized by Genscript, Inc. Each peptide consists of 11 amino acids with the last 3 amino acids of one peptide overlapping with the first 3 amino acids of the next peptide. **Table 2** includes the list of all the peptides used in this study.

**Table 2:** Primary sequences of the BmGPI12 peptides.

BmGPI12 peptide	Amino acid position	Peptide sequence	kDa
P1	50-60	SNPTGAGG**QPN**	1
P2	58-68	**QPN**NEAKK**KAV**	1.2
P3	66-76	**KAV**KLDLD**LMK**	1.3
P4	74-84	**LMK**ETKNV**CTT**	1.3
P5	82-92	**CTT**VNTKL**VGK**	1.2
P6	90-100	**VGK**AKSKL**NKL**	1.2
P7	98-108	**NKL**EGESH**KEY**	1.3
P8	106-116	**KEY**VAEKT**KEI**	1.3
P9	114-124	**KEI**DEKNK**KFN**	1.4
P10	122-132	**KFN**ENLVK**IEK**	1.4
P11	130-140	**IEK**RKKIK**VPA**	1.3
P12	138-148	**VPA**DTGAE**VDA**	1
P13	146-156	**VDA**VDDGV**AGA**	1
P14	154-164	**AGA**LSDLS**SDI**	1
P15	162-172	**SDI**SAIKT**LTD**	1.2
P16	170-180	**LTD**DVSEK**VSE**	1.2
P17	178-188	**VSE**NLKDD**EAS**	1.2
P18	186-196	**EAS**ATEHT**DIK**	1.2
P19	190-200	**TEH**TDIKEKAT	1.3

Overlapping amino acids are indicated by bold letters.

### *Babesia microti* short-term *in-vitro* culture

Short-term *in-vitro *culture of *B. microti* was performed as previously described (Thekkiniath et al., 2018). Under these conditions, the parasite develops from ring to tetrad stages (Lawres et al., 2016) and secretes antigens such as BmGPI12 and BmIPA48, which are also detected in plasma from *B. microti*-infected animals (Thekkiniath et al., 2019). Briefly, one C.B-17.SCID mouse (Cornillot et al., 2016) was infected with the *B. microti* *Lab S1* strain and 50 μL of infected blood (at 15% parasitemia) was mixed with 100 μL of uninfected human RBC (A^+^, 50% hematocrit) and allowed to grow in complete DMEM-F12-based cell culture medium (850 μL) (BE04-687F/U1, Lonza) supplemented with 20% heat-inactivated fetal bovine serum (HI-FBS, F4135, Sigma), 1X-antibiotic/antimycotic solution (15240-062, GIBCO), 2% 50X HT Media Supplement Hybri-Max 230 TM (H0137, Sigma), 1% 200 mM L-Glutamine (25030-081, GIBCO) for 24h at 37°C under hypoxic condition (2% O_2_) (Singh et al., 2022). 1 ml culture volume was centrifuged at 400 x g at room temperature for 10 min, and a 1ml culture supernatant (S) was collected. The cell pellet was further treated with 1% saponin (100 µl) and incubated on ice for 30 min. Hemolysate (H) and parasite pellet (P) fractions were collected after centrifugation at 9,300 x g for 10 min at 4°C. The parasite pellet was resuspended in 30µl of 1X Laemmli sample buffer. The collected S, H, and P fractions were used for immunoblot analysis.

### Immunodetection of BmGPI12

Supernatant (S), hemolysate (H), and parasite pellet (P) fractions from a short-term *in vitro* culture of *B. microti* (iRBCs) and similar fractions from non-infected human RBCs (uRBCs, control) were used in western blots. Sample (S, H and P) were mixed with Laemmli sample buffer (1610747, BIO-RAD), boiled at 80°C for 5 min, separated on 12% mini-protean gels, and transferred onto nitrocellulose membranes. The membrane was blocked with 5% non-fat skim milk (AB10109-01000, American Bio) in Tris-buffered saline (TBS) containing 0.02 M Tris base, 0.15 M NaCl, and 0.1% Tween 20 (1X TBST) for 1h at room temperature followed by incubation with 18 anti-BmGPI12 MAbs (1:250 dilution in blocking buffer) at 4°C overnight. Following treatment with primary antibodies, the membranes were washed three times with 1X TBST (10 min each) and incubated with HRP-conjugated goat anti-mouse IgG (1: 5,000 dilution) (31466, Thermo Fisher) for 1h at room temperature. Subsequently, the membranes were washed twice with 1X TBST and once with 1X PBS and developed using Super signal™ West Pico PLUS chemiluminescent substrate (34577, Thermo Scientific). The blot was imaged using LI-COR Odyssey-Fc imaging system.

### Immunofluorescence assay

Thin blood smears from uninfected and *B. microti*-infected C.B-17.SCID mice RBCs were prepared on glass slides (640-001T, DOT scientific) and fixed with chilled methanol (9070-05, JT Baker) for 15 min at -20°C. The smears were air-dried and blocked in 3% BSA in PBS buffer (A9418, Sigma) for 1h at room temperature. Following this step, the smears were incubated with the mouse monoclonal anti-BmGPI12 antibody 5C11 at 1:500 dilution for 1h at room temperature. This was followed by three washes in 1X PBS containing 0.05% Tween and three washes in 1X PBS, 5 min each. Subsequently, the smears were incubated with goat anti-mouse IgG antibodies conjugated to Alexa Fluor 488 (1:500 dilution) (A-11001, Life Technologies) for 1h at room temperature. This was followed by three washes in 1X PBST and three washes in 1X PBS. The smears were further incubated with Phycoerythrin (PE)-conjugated anti-mouse TER-119 (1:200 dilution) (116207, BioLegend) for 1h at room temperature followed by three washes in 1X PBST and three washes in 1X PBS. Coverslips were then mounted on glass slides using Vectashield mounting medium containing DAPI (H-1200-10, Vector Laboratories) and examined using a Nikon ECLIPSE TE2000-E microscope. A 100X oil immersion objective was used for image acquisition. Excitation at 465-495 nm was used to detect Alexa Fluor 488 positive cells; excitation at 510-560 nm was used to detect PE positive cells, and excitation at 340-380 nm was used to detect DAPI positive cells. The images were acquired using MetaVue with 1,392 x 1,040 pixel as the chosen image size and subsequently analyzed using ImageJ.

### Immunoelectron microscopy

Sample preparation, immune labeling and image processing for immunoelectron microscopic analysis of *B. microti LabS1*–infected mouse (CB.17-SCID) red blood cells (mRBCs) were performed as previously described by Thekkiniath et al. (Thekkiniath et al., 2019). Briefly, *B. microti*-infected mRBCs were fixed in 4% PFA and frozen using a Leica HMP100 at 2,000 psi. The frozen samples were then freeze-substituted using a Leica Freeze AFS unit starting at −95°C using 1% osmium tetroxide, 1% glutaraldehyde, and 1% water in acetone for 10h, warmed to −20°C for 12h and then to 4°C for 2h. The samples were rinsed in 100% acetone and infiltrated with Durcupan resin (Electron Microscopy Science) and baked at 60°C for 24h. Hardened blocks were cut using a Leica UltraCut UC7, and 60-nm sections were collected on formvar/carbon–coated nickel grids. Resin sections were incubated with anti-BmGPI12 monoclonal antibodies at 1: 100 dilution (overnight), rinsed in buffer, and then incubated with the secondary antibody 10 nm protein A gold (UtrechtUMC) for 30 min. The grids were rinsed and fixed using 1% glutaraldehyde for 5 min, rinsed well in distilled water, and contrast-stained using 2% uranyl acetate and lead citrate. The grids were viewed in an FEI Tencai Biotwin TEM at 80 kV. Images were taken using Morada CCD and iTEM (Olympus) software.

### Dot blot analysis of BmGPI12

Dot blot analysis was used to detect specific interactions of the various MAbs with the sub-fragments of BmGPI12 or individual peptides (11 amino acids each). Samples (100 ng) were spotted on a nitrocellulose membrane and dried at room temperature for 1h. The membrane was then blocked by application of 200 µl of blocking buffer (5% BSA in TBS with 0.05% Tween-20 (TBST-0.05%)) (Singh et al., 2022) into each well for 1h at room temperature. Following this step, the blocking buffer solution was replaced with 200 µl of 0.1% BSA in TBST-0.05% buffer and kept for 1h at room temperature or overnight at 4°C. Primary antibodies were diluted 1:1000 in 0.1% BSA in TBST-0.05% buffer and added to the membranes for 1h at room temperature, followed by incubation with HRP-conjugated goat anti-mouse IgG (1: 5,000 dilution) (31466, Thermo Fisher) secondary antibody for 45 min at room temperature. The blots were washed three times with TBST-0.05%, once with PBS (5 min), and then developed using the Super signal™ West Pico PLUS chemiluminescent substrate (34577, Thermo Scientific) and imaged using the Odyssey Fc imaging system.

### Detection of anti-BmGPI12 antibodies in sera or plasma samples from *B. microti* infected Balb/c mice and field mice

Immunoreactivity of mouse plasma or sera to full length BmGPI12 or its sub fragments (F1, F2, F3, F4 and F5) were determined by indirect ELISA. Recombinant BmGPI12 or sub-fragments were coated (50 ng/well) in 96-well Nunc Maxisorp plates and incubated overnight at 4°C. Plates were then blocked with 5% BSA in PBS containing 0.05% Tween 20 (PBST) for 1.5h at 37°C. All sera and plasma dilutions were made in 1% BSA in PBST. Following this step, plates were incubated with sera and plasma from *B. microti*-infected or uninfected mice at 1: 200 dilution for 1.5h at room temperature. For studies involving sera collected from field mice, similar antigen coated plates were incubated with CDC-confirmed *B. microti* PCR positive or negative mouse sera at 1: 200 dilution. Following plasma/sera incubation, plates were washed 4 times with PBST, then incubated for 1h with goat anti-mouse HRP conjugated secondary antibody (1: 5000) (31466, Thermo Fisher). The plates were then washed 4 times with PBST and 100 μL TMB substrate (3,3,5,5,-tetramethylbenzidine (KPL 5120-0083, SeraCare Life Sciences) was added to each well. After a 5 min incubation at room temperature (in the dark), the reaction was stopped by adding 100 μl of 0.1N HCl. Optical density (OD) was measured at 450 nm on a BioTek PowerWave HT plate reader. Standard Deviation (SD) was calculated using Graphpad Prism 9.3 software. The cut-off OD_450_ was determined as mean + 2 x SD of uninfected/negative samples.

### Detection of BmGPI12 in human plasma samples

Plasma samples from blood collected in EDTA from individuals that were either negative or positive for *B. microti* nucleic acid by PCR or transcription-mediated amplification (TMA) were obtained as de-identified samples from the American Red Cross (ARC) under an approved agreement (Tonnetti et al., 2020). 96-well Nunc Maxisorp plates were coated with purified BmGP12-Full length (FL) or sub-fragments F1, F2, F3, F4 or F5 at 50 ng/well (diluted in coating buffer (20 mM Tris-HCl pH 8.5 + 0.1M NaCl)), overnight at 4°C. Wells with only coating buffer were used as controls. Antigen coated wells were blocked with 200 μl ChonBlocking sample dilution ELISA Buffer (9068, Chondrex, Inc.) for 2h at 37°C. Following blocking, *B. microti* positive or negative plasma samples added at 1:250 dilution in 1% BSA in PBST for 1.5h at room temperature. The plates were subsequently washed four times with PBST and incubated with HRP-conjugated Fc gamma fragment specific goat anti-human IgG (32935, Cell Signaling) at 1:10,000 dilution for 1h at room temperature. After four washes with PBST, 100 μL of TMB substrate was added to each well and incubated for approximately 5 min at room temperature in the dark. The reaction was stopped by adding 100 μl of 0.1N HCl. The OD was measured at 450 nm using Bio-Tek Power Wave HT plate reader. Each experiment was independently repeated with two technical replicates each. Standard Deviation (SD) was calculated using Graphpad Prism 9.3 software. The cut-off OD_450_ value was determined as mean + 2 x SD of the OD_450_ value of the negative/uninfected samples.

### mGPAC assay with monoclonal antibody combinations

Sandwich ELISA using monoclonal antibody combinations to detect *B. microti* infection in mice were performed as previously described (Gagnon et al., 2022) with slight modifications. Briefly, 96-well plates were coated with 200 ng/well of capture MAbs (2H6, 1E11, and 4C8), and incubated at room temperature for 2h. The wells were then blocked with 5% BSA in PBST (0.05% Tween-20) and incubated at room temperature for 1h. Following this, plates were incubated overnight at 4°C with either recombinant BmGPI12 (100 ng/well) or with heat inactivated (30 min at 56°C) plasma from uninfected or *B. microti* (LabS1) infected C.B-17.SCID mouse at 1:100 dilution. The plates were then washed 4 times with PBST followed by addition of 2 μg/ml biotinylated detection MAbs (2H6, 1E11, 4C8, 5C11, or 1A5) and incubated at room temperature for 2h. The plates were then washed with PBST and incubated with HRP conjugated streptavidin (KPL 474-3000, SeraCare Life Sciences) at 1:5000 dilution for 1h at room temperature. Finally, the plates were washed four times with PBST, and 100 μl TMB substrate was added, incubated for 5 min at room temperature and 100 μl of 0.1N HCl was added to stop the reaction. The optical density at 450 nm was measured on a BioTek PowerWave HT plate reader. Results were analyzed using the Graphpad Prism 9.3 software and statistical significances were calculated using two-way ANOVA.

The specificity of the MAb combinations was evaluated by using culture supernatants from *B. duncani WA-1* (25% parasitemia), *B. divergens Rouen87* (10% parasitemia), *P. falciparum* 3D7 (10% parasitemia), and *P. falciparum* HB3 (10% parasitemia)-infected human erythrocytes as well as heat inactivated plasma from uninfected or *B. duncani-*infected C3H/HeJ mice (34% parasitemia). *B. duncani* and *B. divergens* were propagated *in vitro* in complete DMEM-F12 (cF12) (Singh et al., 2022) whereas *P. falciparum* strains were propagated in complete RPMI (cRPMI) medium (El Bissati et al., 2006). Plasma from *B. microti* infected or uninfected SCID mice were used as positive and negative controls, respectively. Statistical significances were calculated using one way ANOVA test using the Graphpad Prism 9.3 software. For each sample type the cut-off OD_450_ value was determined as the mean + 2 x SD of the OD_450_ value of the corresponding uninfected sample. Any signal above the cut-off limit was considered a positive result.

## Results

### BmGPI12 cellular distribution in *B. microti*-infected erythrocytes using specific monoclonal antibodies

**Figure 1:**
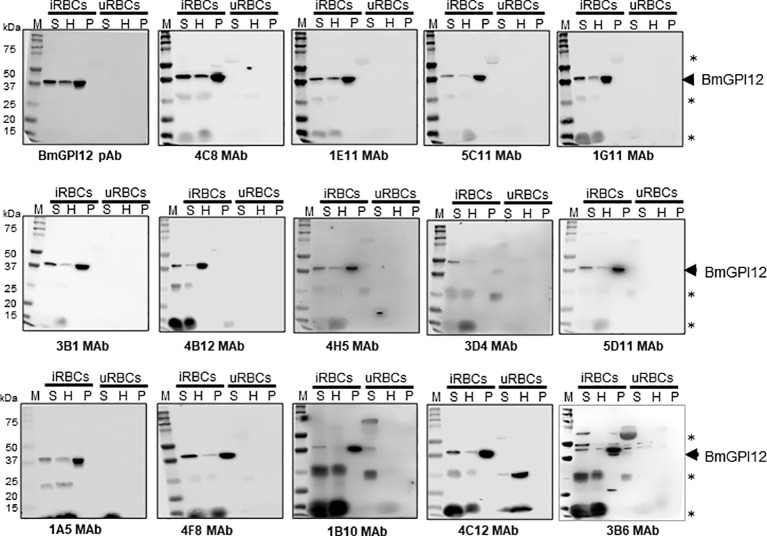
Detection and localization of BmGPI12 using anti-BmGPI12 polyclonal and monoclonal antibodies. Immunoblot analysis of secreted (S), hemolysate (H) and parasite (P) fractions isolated from *B microti* short-term *in vitro* culture (iRBC) or uninfected human RBCs (uRBCs) using α-BmGPI12 polyclonal (pAb) and different monoclonal antibodies (MAbs). Samples were analyzed on a 12% mini protein gel, transferred to nitrocellulose membrane and probed with BmGPI12 poly or monoclonal antibodies as indicated. * indicates nonspecific bands or degradation products.

**Figure 2:**
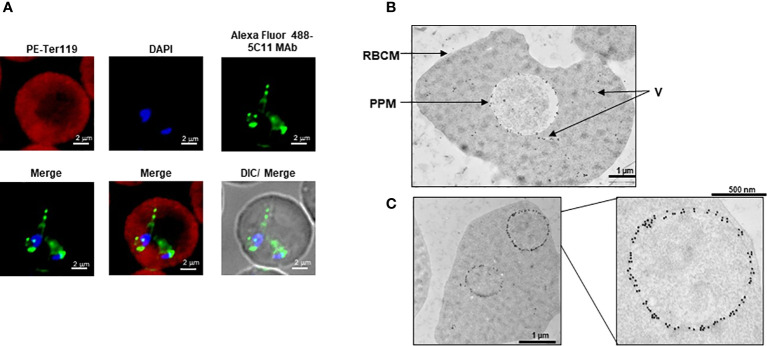
Subcellular localization of BmGPI12. **(A)** Immunofluorescence staining of BmGPI12 using 5C11 monoclonal antibody (raised in mouse) followed by staining with Alexa Fluor (AF)-488 conjugated anti-rabbit secondary antibody (AF488-BmGPI12 pAb, Green) in *B microti*-infected mouse RBCs (Bm-iRBC). Mouse RBCs were stained with Phycoerythrin (PE)-conjugated anti-mouse Ter 119 (PE-Ter119, Red). DAPI (Blue) was used to label parasite DNA. Bars, 2µm. **(B, C)** Transmission immunoelectron microscopic analysis of *B microti* LabS1–infected mouse red blood cells (mRBCs). Ultrathin sections of high-pressure frozen and Durcupan resin–embedded infected RBCs were immunolabeled with anti-BmGPI12 monoclonal antibody (5C11) coupled to 10 nm gold particle. Portion of image **(C)** is magnified to show enlarged parasite showing localization of BmGPI12 in the parasite plasma membrane (PPM). RBCM: red blood cell membrane; V, vesicles. Bars, 1µm and 500 nm.

Eighteen monoclonal antibodies (MAbs) developed against BmGPI12 were recently characterized by ELISA using recombinant BmGPI12 as a target antigen (Gagnon et al., 2022) (**Table 1**). The specificity of these antibodies was assessed by immunoblotting using supernatant (S), hemolysate (H) and free parasites (P) fractions from *B. microti*-infected erythrocytes. Fourteen of the eighteen MAbs recognized the native BmGPI12 in all three fractions (**Figure 1**); the remaining four MAbs, 2A7, 2H6, 3A12, and 4E1, showed weak signal in this analysis. As a control, no signal could be detected using similar fractions from uninfected erythrocytes (**Figure 1**). Immunofluorescence microscopy analyses using these MAbs identified 16 MAbs, which were specific and presented a localization profile for BmGPI12 (**Supplementary Figure S1**; **Figure 2A**) identical to that previously reported using polyclonal antibodies raised against the full-length protein (Thekkiniath et al., 2019). As shown in **Figure 2A**, BmGPI12 could be detected using MAb 5C11 in *B. microti*-infected erythrocytes both on the parasite plasma membrane and the vesicular network produced by the parasite and exported into the erythrocyte cytoplasm (Thekkiniath et al., 2019). Five of the monoclonal antibodies (1G11, 1E11, 5C11, 4C8 and 1A5) were further used in immunoelectron microscopy (IEM) analyses. However, only 5C11 showed a specific signal by IEM in *B. microti*-infected erythrocytes with BmGPI12 localized to the plasma membrane of *B. microti* as well as to the surface of the secreted vesicles (**Figures 2B, C**) as previously reported using polyclonal antibodies (Thekkiniath et al., 2019)

### Anti-BmGPI12 MAbs map to highly immunogenic regions of the secreted antigen

**Figure 3:**
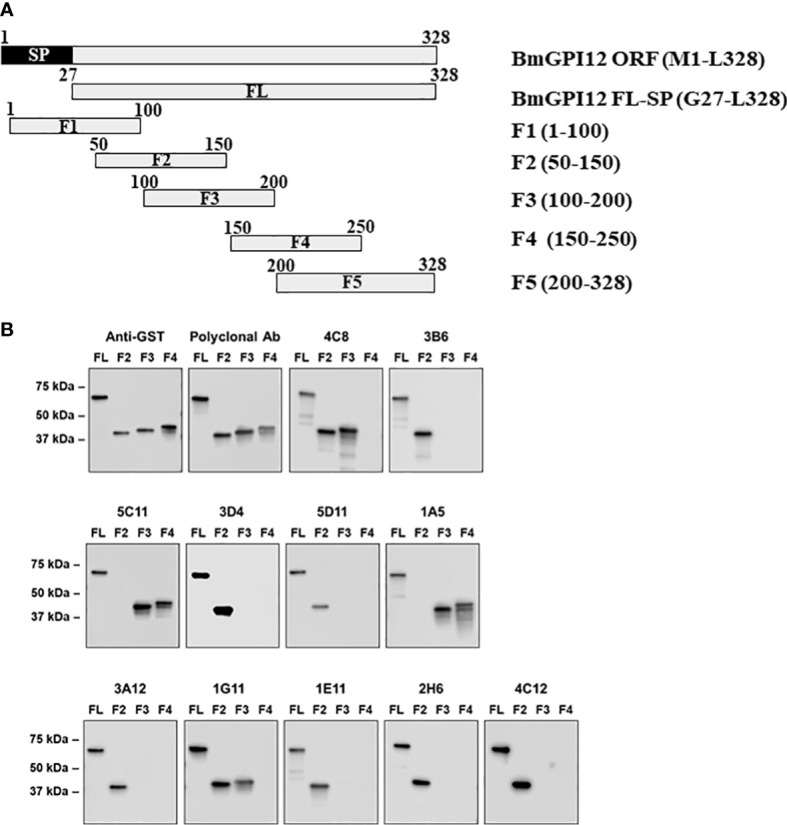
Mapping of MAb binding to specific BmGPI12 fragments. **(A)** Schematic diagram of BmGPI12 and its sub-fragments. **(B)** Western blot analysis using anti-GST or different anti-BmGPI12 MAbs against full length (FL) BmGPI12 or sub-fragments F2, F3 and F4.

**Figure 4:**
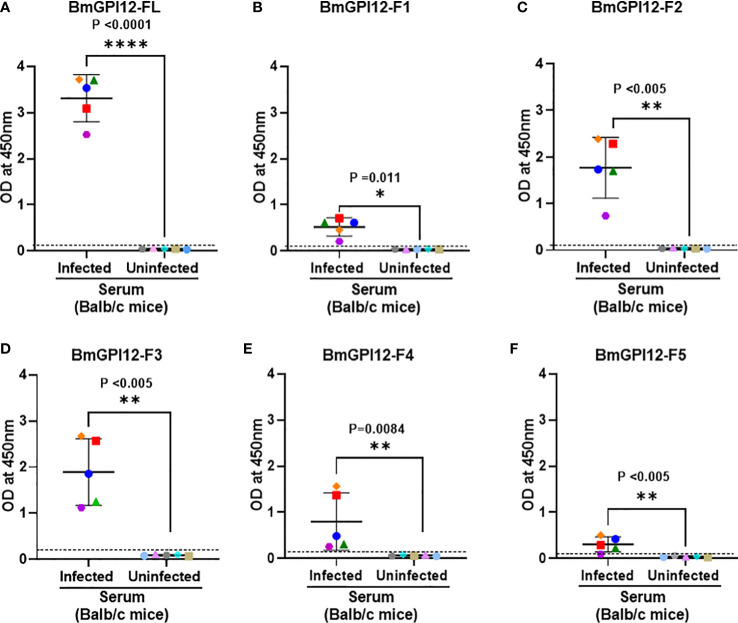
Detection of full length BmGPI12 or its sub-fragments by indirect ELISA using uninfected or *B microti*-infected mouse sera. Heat inactivated sera from *B microti*-infected or uninfected Balb/c mice were used (1:200 dilution) to detect recombinant BmGPI12-FL or sub-fragments F1, F2, F3, F4 or F5 coated on ELISA plates at 50 ng/well. In all graphs, one color represents the reactivity of each mouse serum sample to different BmGPI12 antigens- FL **(A)**, F1 **(B)**, F2 **(C)**, F3 **(D)**, F4 **(E)** and F5 **(F)**. Each assay was conducted more than once with at least two technical replicates per assay. Error bars denote the standard deviation (SD) calculated using the Graphpad Prism 9.3 software. OD_450_ cut-off (dashed line) was determined as the mean + 2 x SD of the OD_450_ value of the uninfected samples. ****: statistically significant (p<0.0001, by two-way ANOVA); **: statistically significant (p<0.005, by two-way ANOVA); *: statistically significant (p=0.011, by two-way ANOVA).

**Figure 5:**
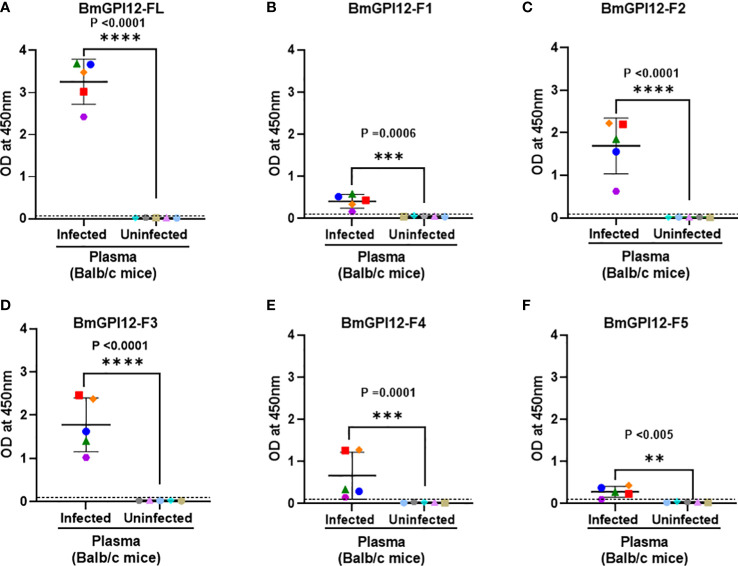
Detection of full length BmGPI12 or its sub-fragments by indirect ELISA using uninfected or *B microti*-infected mouse plasma. Heat inactivated plasma from *B microti*-infected or uninfected Balb/c mice were used (1:200 dilution) to detect recombinant BmGPI12-FL or sub-fragments F1, F2, F3, F4 or F5 coated on ELISA plates at 50 ng/well. In all graphs, one color represents the reactivity of each mouse plasma sample to different BmGPI12 antigens- FL **(A)**, F1 **(B)**, F2 **(C)**, F3 **(D)**, F4 **(E)** and F5 **(F)**. Each assay was conducted more than once with at least two technical replicates per assay. Error bars denote the standard deviation calculated using the Graphpad Prism 9.3 software. OD_450_ cut-off (dashed line) was determined as the mean + 2 x SD of the OD_450_ value of the uninfected samples. ****: statistically significant (p<0.0001, by two-way ANOVA); ***: statistically significant (p<0.0006, by two-way ANOVA); **: statistically significant (p<0.005, by two-way ANOVA).

**Figure 6:**
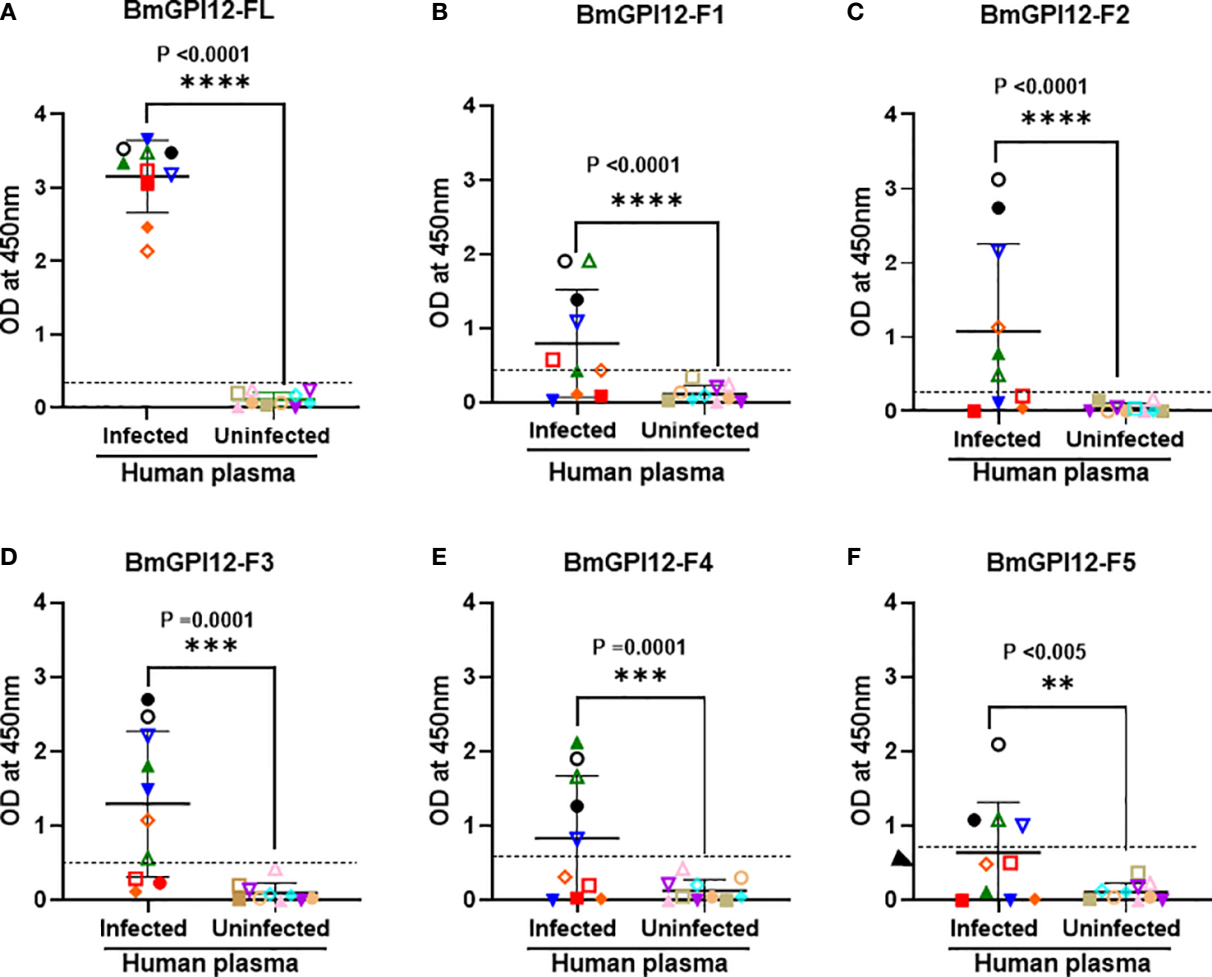
Detection of recombinant BmGPI12 or its sub-fragments using plasma from *B microti* PCR-positive and -negative humans. Graphs show the detection of full length BmGPI12 or its sub fragments (F1-F5) with *B microti* positive (by PCR or TMA) or negative (by TMA) human plasma samples. ELISA plates were coated with 50 ng/well of BmGPI12 FL **(A)** or fragments F1 **(B)**, F2 **(C)**, F3 **(D)**, F4 **(E)**, F5 **(F)**. Human plasma samples were used at 1:250 dilution. Each assay was conducted more than once with at least two technical replicates per assay. Error bars denote the standard deviation(SD) calculated using the Graphpad Prism 9.3 software. In all graphs, each color/symbol represents the reactivity of individual human plasma sample to different BmGPI12 antigens. Statistical significance was calculated using two-way ANOVA. The OD_450_ cut-off (dashed line) denotes the mean + 2 x SD of the OD_450_ value of the negative samples. ****: statistically significant (p<0.0001, by two-way ANOVA); ***: statistically significant (p=0.0001, by two-way ANOVA); **: statistically significant (p<0.005, by two-way ANOVA).

To identify the specific regions in BmGPI12 that are recognized by the various monoclonal antibodies, the full-length protein (FL: amino acids 28-328) as well as five overlapping fragments F1 (amino acids 1 to 100 of BmGPI12), F2 (amino acids 50 to 150 of BmGPI12), F3 (amino acids 100 to 200 of BmGPI12), F4 (amino acids 150 to 250 of BmGPI12) and F5 (amino acids 200 to 328 of BmGPI12) were purified as recombination fusion proteins using an N-terminal GST tag and used in dot blot and immunoblot assays with each of the 18 MAbs. A schematic representation of the BmGPI12 FL and sub-fragments are shown in **Figure 3A**. As shown in **Figure 3B**, 13 MAbs were found to bind to either a specific fragment or 2 overlapping fragments of BmGPI12. Monoclonal antibodies 5D11, 2H6, 3D4, 3B6, 4C12, 3A12, and 1E11 specifically recognized the F2 fragment; 4C8, and 1G11 mapped specifically to both F2 and F3 fragments; whereas 5C11 and 1A5 MAbs mapped specifically to the F3 and F4 fragments. The F1 and the F5 fragments showed low reactivity with the MAbs in immunoblot and dot blot (data not shown). The antigenicity profile of the F1, F2, F3, F4 and F5 fragments of BmGPI12 were further characterized using serum and plasma samples from uninfected and *B. microti*-infected mice as well as human plasma from *B. microti* positive or -negative individuals in indirect ELISA. As shown in **Figures 4A–F**, **5A–F**, both sera and plasma from *B. microti*-infected mice reacted strongly with the recombinant antigens with optimal immunoreactivity achieved with the full length BmGPI12 (OD at 450 nm ranged between 2.5 and 3.7) followed by the F2 and F3 fragments whereas the F4 fragment showed only a modest reactivity. As a control, no reactivity could be found using sera and plasma from uninfected mice (**Figures 4A–F**, **5A–F**). Similarly, the immunoreactivity of human plasma samples from *B. microti* positive individuals reacted with all the fragments of BmGPI12 with the highest reactivity recorded with fragments F2 and F3 (**Figures 6A–F**). No reactivity could be detected with plasma from *B. microti*-negative individuals (**Figure 6**). Both plasma and serum samples from mice and humans that showed the strongest immunoreactivity using the full length recombinant BmGPI12 were also those that reacted strongly with the F2 and F3 fragments.

**Figure 7:**
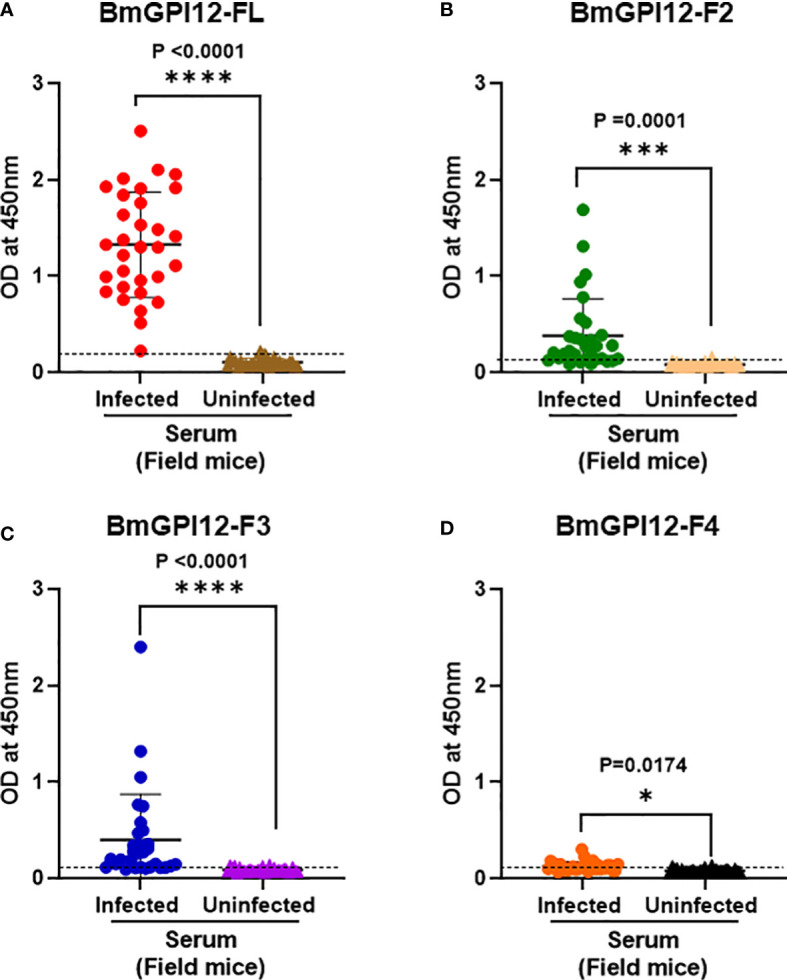
Immunodetection of recombinant BmGPI12 or sub-fragments using serum samples from wild mice. ELISA plates were coated with recombinant BmGPI12- full length (FL) **(A)**, sub-fragments F2 **(B)**, F3 **(C)** and F4 **(D)** at 50 ng/well. Graphs represent immunoreactivity of *Babesia* positive and negative wild mice sera (used at 1:200 dilution) to respective coated antigens. Red, green, blue and orange solid circles represent responses of *B microti* PCR positive samples to recombinant BmGPI12-FL, F2, F3 and F4 sub-fragments respectively. Brown, peach, purple and black solid triangles represent responses of *B microti* PCR negative samples to recombinant BmGPI12- FL, F2, F3 and F4 sub-fragments respectively. Each assay was conducted more than once with at least two technical replicates per assay. Error bars denote the standard deviation calculated using the Graphpad Prism 9.3 software. The OD_450_ cut-off (dashed line) denotes the mean + 2 x SD of the OD_450_ value of the negative samples. ****: statistically significant (p<0.0001, by two-way ANOVA); ***: statistically significant (p=0.0001, by two-way ANOVA); *: statistically significant (p=0.017, by two-way ANOVA).

To further assess whether a similar immunoreactivity profile could be seen in reservoir animals, we screened 62 serum samples obtained from field mice from various regions in Connecticut, USA. These samples were previously characterized by PCR to detect *B. microti* 18S rDNA and separated into 31 PCR-positive and 31 PCR-negative samples. As shown in **Figures 7A–D**, the immunoreactivity of the majority of *B. microti* PCR-positive samples were found to be above the OD_450_ cut-off value of the full-length antigen (>97%) as well as the F2 (80%), and F3 (71%) fragments (p values ≤ 0.0001 by 2-way ANOVA). The cut-off OD_450_ value was determined as the mean + 2 x SD of the OD_450_ value of the corresponding negative/uninfected sera samples for each antigen. Consistent with the PCR data, all sera from PCR-negative field mice showed no signals above the OD_450_ cut-off (**Figure 7**).

### Epitope mapping of MAbs to BmGPI12

**Figure 8:**
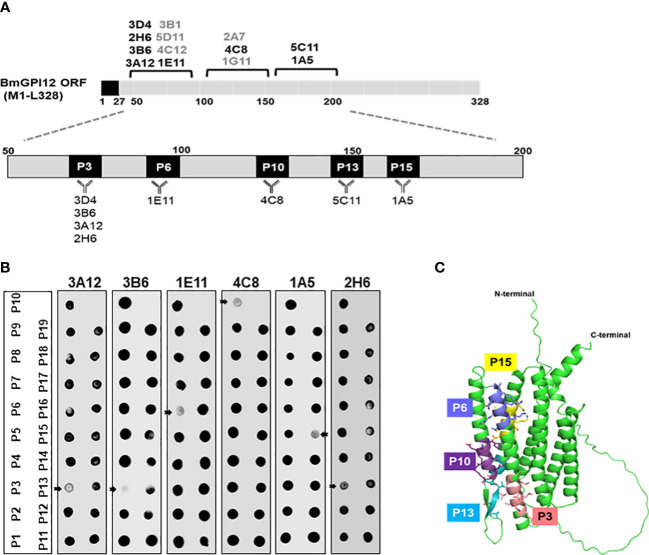
Epitope mapping of BmGPI12 monoclonal antibodies using overlapping peptides and localization of the peptides on the predicted BmGPI12 structure. **(A)** Schematic representation of the binding sites of 13 monoclonal antibodies to the BmGPI12 antigen. The specific 11-amino acid binding sites (P3, P6, P10, P13 and P15) recognized by the 8 monoclonal antibodies on BmGPI12 are highlighted. **(B)** Panels represent dot blot-based competition assays used to examine the interaction between the BmGPI12 antigen and various monoclonal antibodies in the presence of each of the overlapping nineteen 11-amino acid peptides (P1-19). The panel shows the analysis for 3A12, 3B6, 1E11, 4C8, 1A5 and 2H6 monoclonal antibodies. Arrows indicate where specific peptides block the binding of the MAb to the antigen. **(C)** Predicted three-dimensional structure of BmGPI12 and the location of the P3, P6, P10, P13 and P15 peptides.

The finding that the F2, F3, and F4 fragments of BmGPI12 are the most immunogenic regions of the antigen led us to investigate the exact binding sites of the monoclonal antibodies by epitope mapping (**Figure 8A**). A set of 19 small overlapping peptides (11 amino acids in length with 3 amino acid overlaps between adjacent peptides) covering the F2 to F4 fragments of the BmGPI12 antigen were synthesized (**Table 2**) and coated into nitrocellulose membranes. Specific interactions of the peptides with the 13 MAbs were determined using a dot blot-based direct binding. The specific binding of MAbs to individual peptides was further validated by examining the ability of the peptides to interfere with the binding of MAbs to the F2, F3 and F4 fragments (**Figures 8A, B**). As shown in **Figure 8B**, peptide P3 effectively blocked the binding of the F2 fragment to 3A12, 3B6, 3D4, and 2H6 MAbs; peptides P6 and P10 blocked the binding of F2 domain to 1E11 and 4C8 MAbs, respectively; and peptide P15 blocked the binding of the F3 domain to 1A5 MAb. Taken together, 3A12, 3B6, 3D4 and 2H6 MAbs mapped to P3 (KAVKLDLDLMK, BmGPI12^66-76^); 1E11 MAb to P6 (VGKAKSKLNKL, BmGPI12^90-100^); 4C8 MAb to P10 (KFNENLVKIEK, BmGPI12^122-^132), 5C11 MAb to P13 (VDAVDDGVAGA, BmGPI12^136-156^); and 1A5 MAb to P15 (SDISAIKTLTD BmGPI12^162-172^) (**Figure 8A**; **Table 3**).

**Table 3:** Primary sequences of the 5 BmGPI12 peptides recognized by a set of monospecific monoclonal antibodies.

BmGPI12 peptide #	Amino acid position	Peptide sequence	Monoclonal Antibodies
			3D4
			3B6
P3	66-76	KAVKLDLDLMK	3A12
			2H6
P6	90-100	VGKAKSKLNKL	1E11
P10	122-132	KFNENLVKIEK	4C8
P13	146-156	VDAVDDGVAGA	5C11
P15	162-172	SDISAIKTLTD	1A5

To gain further insights into the immunogenicity profile of BmGPI12 at the structural level, the 3-dimensional structure of protein was generated using the AlphaFold Protein Structure Database (https://alphafold.com/entry/A0A0K3AT66) (**Figure 8C**). While the N-terminal 58 amino acids (including 27 amino acids of the cleaved signal peptide sequence) and the C-terminal 19 amino acids were found to be unstructured, most of the sequences between the two extremities were modeled with high accuracy scores. The predicted protein structure showed 2 distinct bundles of helices, one with 3 helices in the N-terminal region and the other with 4 helices in the C-terminal region. The N-terminal 3 helices encompass the F2 and the F3 fragments consistent with their highly immunogenic profile. Interestingly, peptides P3, P6, P10, P13 and P15, which are within these 2 fragments, were found to be on the surface of the protein with P3, P6, P10, and P13 directly exposed to the surface of the helices (**Figure 8C**), making them easily accessible to antibody binding.

### Specificity of ELISA assays based on anti-BmGPI12 MAbs

**Figure 9:**
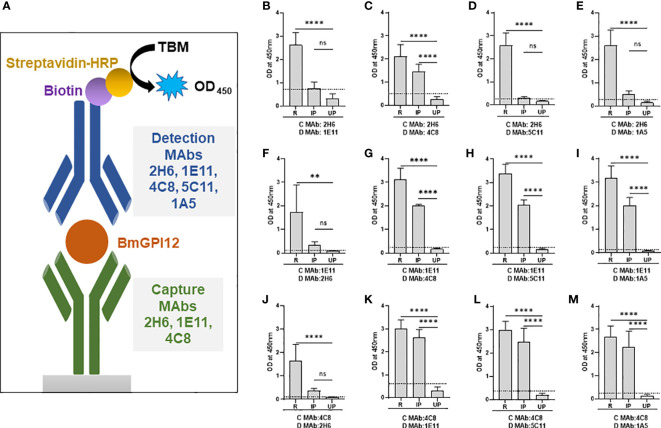
Monoclonal antibody combinations for detection of recombinant and native BmGPI12. **(A)** Schematic representation of the mGPAC antigen capture assay. MAb, monoclonal antibody; TMB, 3,3′,5,5′-Tetramethylbenzidine; HRP, Horseradish Peroxidase; OD_450_, Optical Density at 450 nm. **(B-M)** mGPAC antigen capture ELISA was performed with different combinations of 2H6, 1E11, 4C8, 5C11 and 1A5 as either capture or detection antibodies. Assays were conducted using either recombinant BmGPI12 (R), plasma from uninfected (UP) or *B microti*-infected mice (IP). C: capture antibody; D: detection antibody. Each data set represents the mean of 3 independent experiments each performed in duplicates. Error bars denote the standard deviation calculated using the Graphpad Prism 9.3 software. ****, statistically significant (p<0.0001, by one-way ANOVA); **, statistically significant (p=0.0015, by one-way ANOVA); ns, non-significant. The OD_450_ cut-off (dashed line) denotes the mean + 2 x SD of the OD_450_ value of the uninfected plasma.

**Figure 10:**
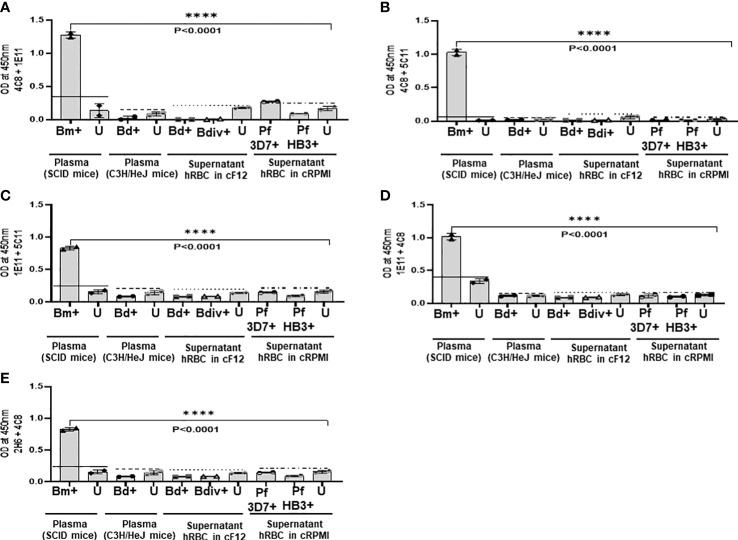
Specificity of the mGPAC assay. Antigen capture assays with different combinations **(A–E)** of anti-BmGPI12 monoclonal antibodies were conducted using culture supernatants from *B duncani WA-1* (25% parasitemia), *B divergens Rouen87* (10% parasitemia), *P. falciparum* 3D7 (10% parasitemia), and *P. f*alciparum HB3 (10% parasitemia)-infected human erythrocytes as well as heat inactivated plasma from uninfected or *B duncani-*infected C3H/HeJ mice (34% parasitemia). Plasma from *B microti* infected or uninfected SCID mice were used as positive and negative controls, respectively. Parasite cultures that were available in the laboratory at the time of the experiments were used. Error bars denote the standard deviation (SD) calculated using the Graphpad Prism 9.3 software. For each sample type, the OD_450_ cut-off (dashed line) denotes the mean + 2 x SD of the OD_450_ value of the corresponding uninfected sample. ****, statistically significant (p<0.0001, by one-way ANOVA). Bm, *B microti*; Bd, *B duncani*; Bdiv, *B >divergens*; Pf, *P. falciparum*; +: infected; U, uninfected; hRBCs, human red blood cells; cF12, complete DMEM-F12; cRPMI, complete RPMI.

Our finding that 4C8, 1E11, 5C11, 1A5 and 2H6 map to specific epitopes on BmGPI12 led us to evaluate specific combinations of monoclonal antibodies that can be used to detect this antigen using antigen capture ELISA assays (mGPAC assay). **Figure 9A** shows the schematic representation of the mGPAC assay that was used to evaluate different monoclonal antibody combinations. All 5 monoclonal antibodies were found to be suitable as detection antibodies, whereas only 2H6, 1E11 and 4C8 were found to be suitable as capture antibodies (**Figure 9**). Whereas all 12 possible combinations of capture and detection antibodies detected the purified recombinant BmGPI12, only 7 of these combinations detected the native secreted antigen in plasma from *B. microti*-infected mice (**Figure 9B**). When using recombinant protein in the capture assay, capture MAb 2H6 worked best with detection MAbs 1E11, 4C8, 5C11, and 1A5 (**Figures 9B–E**), while capture MAb 1E11 was most suitable in combination with detection MAbs 4C8, 5C11 and 1A5 (**Figures 9F–I**). Similarly, 4C8 as a capture antibody worked best with detection MAbs 1E11, 5C11 and 1A5, while the 4C8-2H6 combination showed a comparatively weaker detection (**Figures 9J–M**). When *B. microti*-infected mouse plasma was used as the antigen source, the signal was mostly similar to that obtained for the recombinant protein when capture MAbs 1E11 and 4C8 were used (**Figures 9F–M**). In contrast, the capture MAb 2H6 detected BmGPI12 in the plasma only in combination with 4C8 (**Figures 9C**) but poorly detected the protein when combined with MAbs 1E11, 5C11 or 1A5 (**Figures 9B, D, E**). As a control, no significant signal could be detected with the MAb combinations using plasma from uninfected mice (**Figures 9B–M**). Overall, we identified 7 different combinations of capture and detection antibodies (2H6-4C8, 1E11-4C8, 1E11-5C11, 1E11-1A5, 4C8-1E11, 4C8-5C11, 4C8-1A5) that were optimal for detection of secreted BmGPI12 protein in plasma from *B. microti*-infected mice using antigen capture ELISA. Noteworthy, the combination 1E11+4C8 has recently been validated in a capture assay using plasma samples from *B. microti*-infected humans (Gagnon et al., 2022). The specificity of these antibody combinations was further tested using plasma from *B. duncani*-infected mice as well as supernatants from cultures of other *Babesia* species (*B. duncani* WA-1 isolate and *B. divergens Rouen87* strain) as well as two *Plasmodium falciparum strains* 3D7 and HB3) and we found strong specificity with the following combinations - 2H6-4C8, 1E11-4C8, 1E11-5C11, 4C8-1E11 and 4C8-5C11, where no significant signal above background could be detected (**Figures 10A–E**; **Supplementary Figure S2**).

## Discussion

Mapping the binding sites of antibodies to proteins can be an invaluable tool in various fields including immunology, cellular biology, structural biology and pharmacology by unraveling important cellular processes such as immune signaling, receptor binding and pathogen invasion. Determining the sites of these antibody interactions could also facilitate the development of specific therapeutic and diagnostic strategies to fight pathogens and detect active infections. In this study, we have used this approach to map the specific sites of several monoclonal antibodies that bind to the most important immunogenic regions of the major antigen, BmGPI12, of the human pathogen *B. microti*.

Of 18 monoclonal antibodies characterized in this study, 14 showed strong signals by immunoblotting whereas the other 4 showed weak signal in this assay. On the other hand, only 2 antibodies (3B1 and 3A12) failed to detect the native BmGPI12 antigen by immunofluorescence. While we did not test all the antibodies by immunoelectron microscopy, out of 5 monoclonal antibodies tested (1G11, 1E11, 5C11, 4C8 and 1A5), only 5C11 showed the predicted localization of the antigen to the parasite plasma membrane as well as to the vesicular network produced by the parasite from this membrane as was previously demonstrated using polyclonal antibodies raised against the full length protein (Thekkiniath et al., 2019). The differences in the reactivity of these antibodies in different assays could be due to differences in epitope conformation in the native versus denatured antigen and the types of assays used.

Our strategy to map the specific sites of binding of monoclonal antibodies to BmGPI12 involved first identifying specific regions in the protein that are recognized by these antibodies and then designing overlapping 11 amino acid peptides covering these regions. Using 5 overlapping 100 amino acid sub-fragments (F1-F5) of BmGPI12, we were able to map 14 monoclonal antibodies to specific regions of the protein. Using plasma and sera from uninfected and *B. microti*-infected inbred mice, as well as sera samples from field mice (reservoir animals), we found that F2 and F3 were the most immunogenic fragments of the protein. Our results using sera from *B. microti*-infected field mice showed stronger serum immunoreactivity (97%) to recombinant full-length BmGPI12 protein compared to the F2 (80%) and F3 fragments (71%). This could be attributed to the presence of more epitopes on the full-length protein than on the individual fragments.

We also found that the immunogenic profile of BmGPI12 using sera from *B. microti*-infected field mice (reservoir) varied between individual mice. The differences are likely due to differences in parasitemia in the field mice and the immune response of each mouse to infection.

Our analysis of human plasma samples from *B. microti* positive and negative individuals showed a response profile similar to that observed in mouse. While the range of antibody reactivity varied between the full-length recombinant BmGPI12 and the F2 and F3 fragments, blood samples that displayed the strongest immunoreactivity to the full-length protein were also those that displayed the strongest reactivity to the F2 and F3 fragments. Competition studies using overlapping 11 amino acid peptides further mapped 8 of the anti-BmGPI12 monoclonal antibodies to 5 specific peptides within the highly immunogenic region of the antigen encompassing the F2 and F3 fragments. The predicted three dimensional structure of BmGPI12, which was generated using AlphaFold Protein Structure Database (Jumper et al., 2021; Varadi et al., 2022), showed that the N-terminal triple helix structures encompassing the F2 and F3 fragments contain the majority of the antigenic sites of the protein consistent with the high immunogenicity of this region. Noteworthy, the immunoreactivity of F1 and F4 with plasma from *B. microti*-infected humans was much higher than that from infected mice, highlighting differences between the two mammalian hosts in their response to *B. microti* infection.

While the exact role of BmGPI12 in *B. microti* virulence remains unknown, this protein has been demonstrated to be an excellent biomarker of active infection (Thekkiniath et al., 2018) and is highly conserved among six *B. microti* isolates examined by whole genome sequencing (Silva et al., 2016). Our epitope mapping of a set of BmGPI12 monoclonal antibodies that detect the native secreted antigen with high specificity was critical to our design of optimal antibody combinations for ELISA-based antigen capture assays and rapid detection of active *B. microti* infection. One such combination consisting of two MAbs, 1E11and 4C8, was found to be optimal for detection of *B. microti* infection in human blood (Gagnon et al., 2022). In addition to their use in the detection of active *B. microti* infection, these antibodies may serve as useful resources to understanding the biological function of this important antigen, unraveling its mode of secretion, and determining its host targets.

## Data availability statement

The original contributions presented in the study are included in the article/**Supplementary Material**. Further inquiries can be directed to the corresponding author.

## Ethics statement

The animal study was reviewed and approved by Institutional Animal Care and Use Committees (IACUC) at Yale University.

## Author contributions

MC, J-YC, PS, VK and AP: Investigation, methodology, formal analysis, visualization, writing original draft, review and editing. JG, ST, GG and JT: Investigation, methodology, review and editing. SW: Sample collection and analysis. CB and ML: Conceptualization, supervision, funding acquisition, project administration, writing original draft, review and editing. All authors have read and agreed to the published version of the manuscript. All authors contributed to the article and approved the submitted version.

## Funding

The research described herein was supported by NIH grant A136118 to ML and CB. CB research is also supported by NIH grants AI138139, AI123321, AI152220 and AI153100 and AI136118; the Steven and Alexandra Cohen Foundation (Lyme 62 2020); the Global Lyme Alliance and The Blavatnik Family Foundation. The content is solely the responsibility of the authors and does not necessarily represent the official views of the National Institute of Health.

## Acknowledgments

We thank Isaline Renard for providing blood samples from infected mice to use in some of the assays described in this study, Pratap Vydyam for doing short-term *in-vitro* culture of *B. microti*, Shalev Gihaz for generating the three-dimensional protein structure, Morven Graham and Xinran Liu for help with the IEM analyses, and Sara Mootien for her initial analysis of the monoclonal antibodies used in this study. We would also like to thank Dr. Laura Tonnetti and the American Red Cross for providing the blood samples for these studies.

## Conflict of interest

Authors JG, ST, GG and ML are employed by L2 Diagnostics, United States.

The remaining authors declare that the research was conducted in the absence of any commercial or financial relationships that could be construed as a potential conflict of interest.

## Publisher’s note

All claims expressed in this article are solely those of the authors and do not necessarily represent those of their affiliated organizations, or those of the publisher, the editors and the reviewers. Any product that may be evaluated in this article, or claim that may be made by its manufacturer, is not guaranteed or endorsed by the publisher.
