# Supplementary figures and images for: Epitope profiling of monoclonal antibodies to the immunodominant antigen BmGPI12 of the human pathogen Babesia microti
Source: Front Cell Infect Microbiol. 2022 Nov 25;12:1039197. doi: 10.3389/fcimb.2022.1039197 (PMC9732259; doi:10.3389/fcimb.2022.1039197)

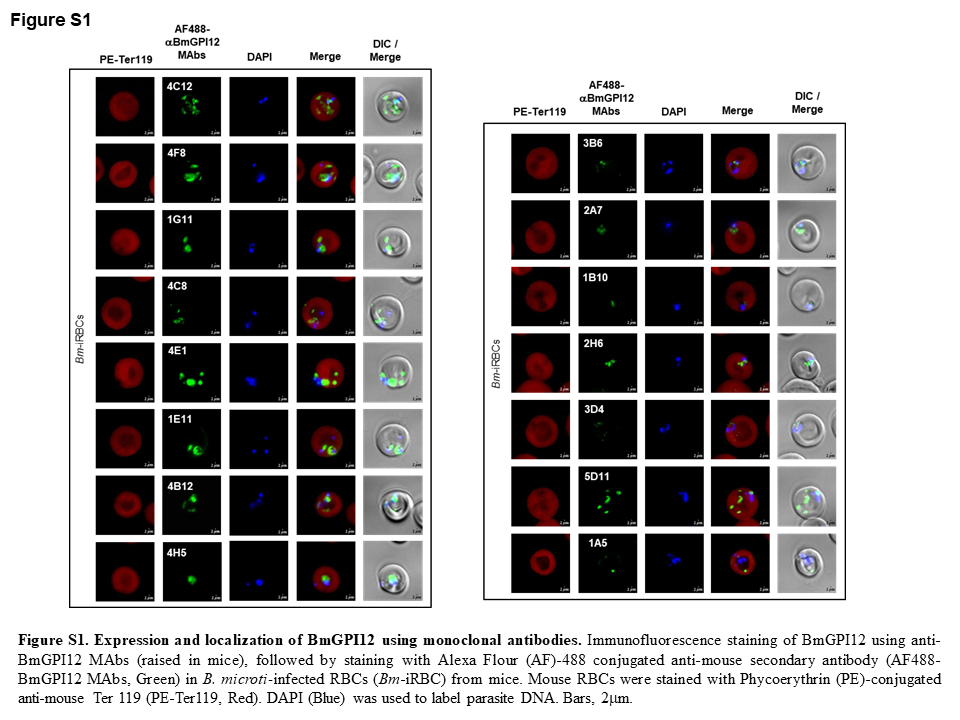

Supplement: Supplementary file 1 [file Image_1.tif]

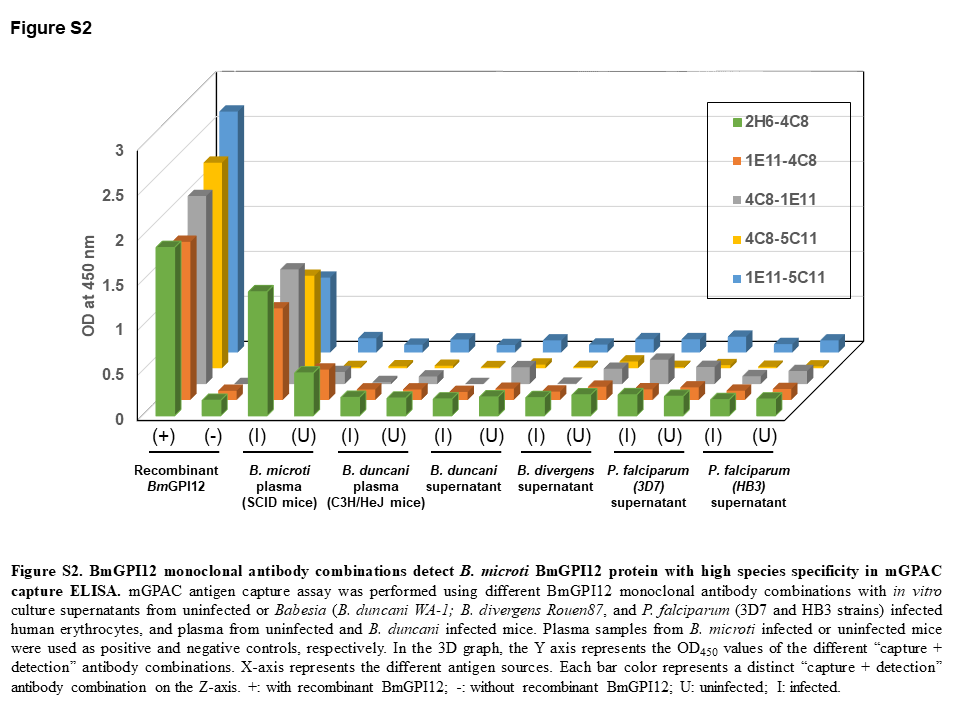

Supplement: Supplementary file 2 [file Image_2.tif]
